# Undiagnosed Symptomatic Hepatic Duplication Cysts and Bilateral Renal Cysts in a 75-Year-Old Female: A Case Report

**DOI:** 10.7759/cureus.78431

**Published:** 2025-02-03

**Authors:** Sariah Watchalotone, Nicholas Smith, Halley McDonald, Luke Lawley, Imtiaz Ahmed

**Affiliations:** 1 Radiology, Midwestern University Arizona College of Osteopathic Medicine, Glendale, USA; 2 Radiology, Tempe St. Luke's Hospital, Tempe, USA

**Keywords:** bilateral renal cysts, congenital malformation, foregut duplication cyst, hepatic duplication cysts, liver cysts

## Abstract

Hepatic duplication cysts are rare congenital malformations resulting in cystic structures within the liver. These lesions are typically diagnosed in pediatric patients and are often asymptomatic. We present the case of a 75-year-old female patient who presented to the emergency department with diffuse abdominal pain. Examination revealed a palpable abdominal mass. Imaging included a CT scan of the abdomen, which revealed multiple hepatic duplication cysts and bilateral renal cysts. This case emphasizes the importance of recognizing rare pathologies such as hepatic duplication cysts, particularly in adult patients. This rare case of co-occurring hepatic duplication cysts and renal cysts may enhance understanding of the clinical presentation, diagnosis, and management of hepatic duplication cysts.

## Introduction

Hepatic duplication cysts are rare congenital malformations that arise during early embryonic development and result in cystic structures within the liver [1,2]. These congenital anomalies, typically identified in the pediatric population, are often asymptomatic and may be susceptible to misdiagnosis due to low incidence and a lack of specific indications for imaging [3]. However, depending on size and location, they may present with symptoms such as abdominal pain, biliary obstruction, nausea, and vomiting [3,4]. On CT imaging, hepatic duplication cysts appear as well-circumscribed, fluid-attenuated lesions without enhancement that may show calcifications and internal septations [5]. Differential diagnoses for focal liver lesions on abdominal imaging include hepatocellular adenoma, focal nodular hyperplasia, hemangioma, polycystic liver disease, uterine and ovarian masses, and cystic renal cell carcinoma [6].

## Case presentation

**Figure 1:**
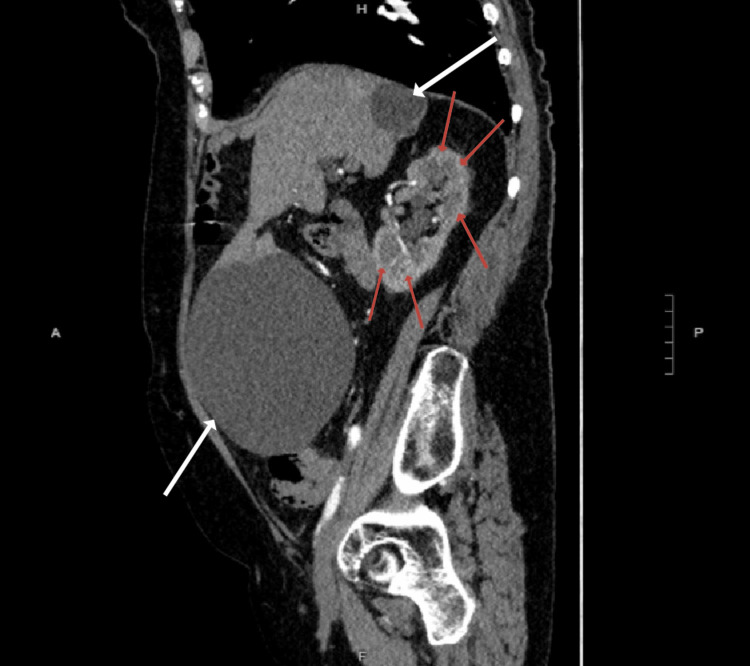
The CT image of the abdomen in the sagittal plane showing numerous renal cysts in the right kidney and large hepatic duplication cysts on the anterior and posterior right lobe of the liver. Red arrows: Renal cysts, White arrows: Hepatic duplication cysts

**Figure 2:**
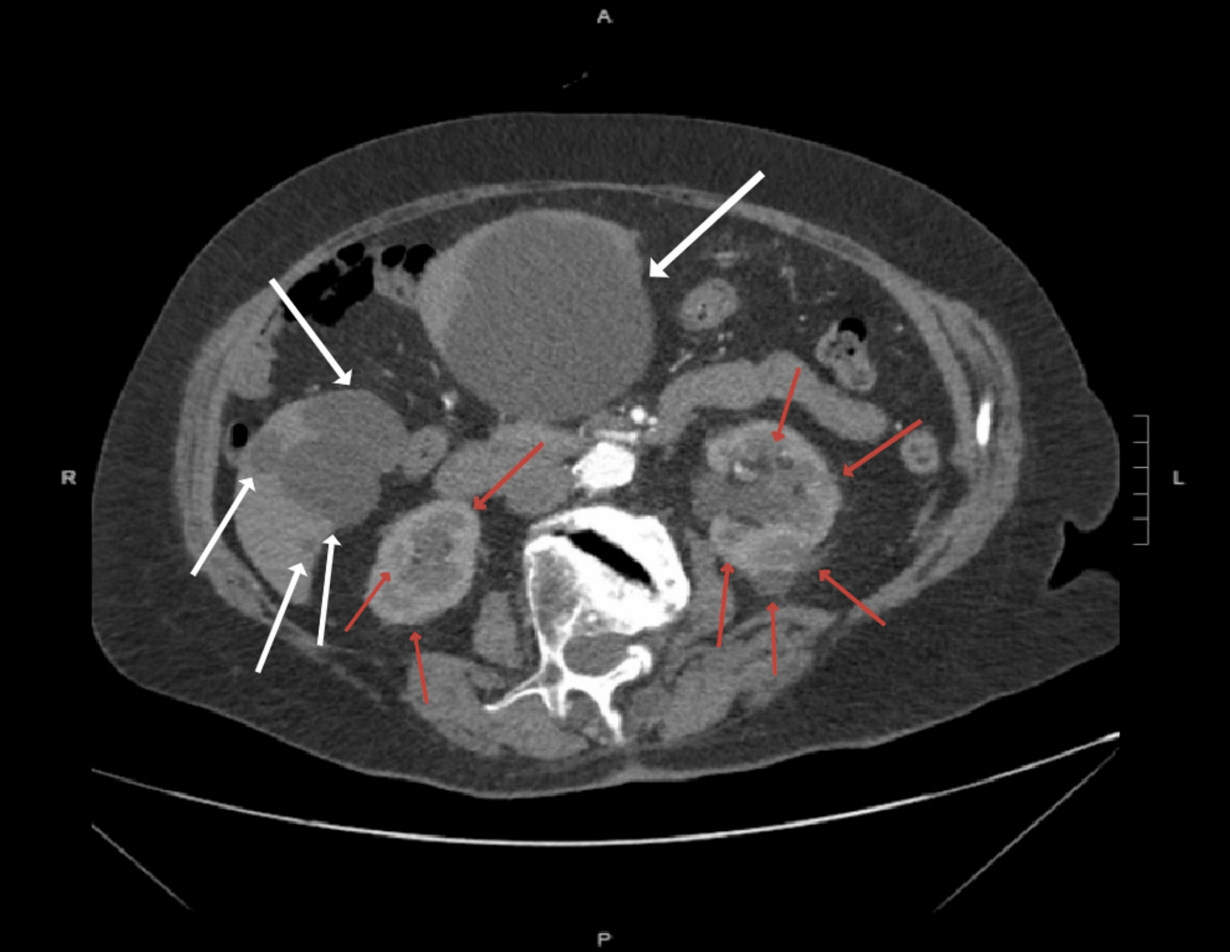
The CT image of the abdomen in the axial plane showing numerous bilateral renal cysts and multiple hepatic duplication cysts. Red arrows: Renal cysts, White arrows: Hepatic duplication cysts

A 75-year-old female presented to the emergency department with a one-week history of diffuse abdominal pain. The patient denied any additional symptoms, including fever, chills, shortness of breath, nausea, vomiting, dysphagia, diarrhea, constipation, bloating, hematuria, urinary urgency, urinary incontinence, or flank pain. The patient denied any pertinent medical or surgical history, prior history of similar episodes of abdominal pain, recent illness, sick contacts, or recent travel. On physical examination, the patient was afebrile with stable vital signs, and a large non-pulsatile mass was noted upon palpation of the abdomen. No abdominal herniation, ascites, or abdominal distension was visualized. Imaging evaluation included CT imaging of the abdomen, which displayed a large midabdominal hepatic duplication cyst measuring 142 x 144 x 123 mm (Figure 1), multiple cysts in the right lobe of the liver, and numerous bilateral renal cysts with no evidence of hydronephrosis or renal stones (Figure 2). The patient was referred to an outpatient gastroenterologist for workup and treatment, including sampling and possible surgical resection of the hepatic duplication cysts.

## Discussion

Enteric duplication cysts are congenital anomalies due to abnormal development of the embryonic foregut [2,7,8]. Cysts are subepithelial lesions composed of tissue native to the involved organ and can occur anywhere along the gastrointestinal tract [2,3,9]. While alimentary tract duplications are estimated to occur in one in every 4500 births, the incidence of hepatic duplications is poorly understood [10]. Typically, lesions are identified in infants and children but may be found incidentally in asymptomatic adults. Patients with enteric duplications may present with a wide array of clinical manifestations depending on the cyst's location, size, and morphology. Symptoms may include dysphagia, vomiting, abdominal mass, abdominal pain, constipation, diarrhea, intussusception, bowel obstruction, torsion, perforation, portal hypertension, or hemorrhage [2-4,8,10,11]. Most lesions are benign, but malignant transformations may occur rarely [8].

Morphology on imaging is dependent on the tissue involved but typically demonstrates lesions that are typically well-circumscribed, anechoic to hypoechoic or low-intensity lesions with possible fluid content, and complicated in nature with possible internal septations or multilocular appearance [9,12]. These imaging findings should warrant further workup to differentiate duplication cysts from other complicated cystic hepatic lesions. Differential diagnoses for hepatic cysts include hemangioma, polycystic liver disease, autosomal dominant polycystic kidney disease, cystadenomas, or cystadenocarcinomas [5,6]. The relationship between renal cysts and hepatic cysts is well established, most notably seen with autosomal dominant polycystic kidney disease [5]. However, no known relationship between hepatic duplication cysts and renal cysts has been identified.

Ultrasound, CT, and MRI modalities are critical in evaluating hepatic lesion morphology to guide appropriate management. A biopsy may be useful in the setting when a definitive diagnosis cannot be made by imaging alone. Special consideration should be made for which hepatic lesions to biopsy, as hepatic adenomas are highly vascularized and pose a bleeding risk [6]. 

Surgical excision is the definitive treatment for symptomatic duplication cysts to prevent complications and reduce the potential for malignant transformation [1,11]. Lesions found incidentally and diagnosed as asymptomatic simple hepatic cysts may be managed conservatively with regular surveillance. It is important to thoroughly differentiate duplication cysts from lesions such as cystadenomas or cystadenocarcinomas due to the risk of malignant transformation. For these reasons, lesion morphology on imaging is critical in diagnosis and for differentiating duplication etiology from other possible conditions [6].

## Conclusions

This case highlights the importance of maintaining a broad differential for patients presenting with nonspecific abdominal symptoms. In this case, the patient’s clinical presentation and age at the onset of symptoms were outside of the typical range for rare pathologies such as hepatic duplication cysts and were particularly unusual due to the co-occurrence of bilateral renal cysts. Hepatic duplication cysts are a rare but important consideration in the diagnosis and management of nonspecific abdominal symptoms, where early recognition is crucial to avoid misdiagnosis and ensure appropriate management. Further research and case studies are needed to better understand the clinical presentation, methods for diagnosis, and optimal treatment strategies for this rare condition, especially in adult patient populations.
